# Retrospective study using biosensor data of a milking Holstein cow with jejunal haemorrhage syndrome

**DOI:** 10.17221/73/2023-VETMED

**Published:** 2023-09-28

**Authors:** Seungmin Ha, Seogjin Kang, Mooyoung Jung, Eunjeong Jeon, Seongsoo Hwang, Jihwan Lee, Jongho Kim, You-Chan Bae, Jinho Park, Ui-Hyung Kim

**Affiliations:** ^1^National Institute of Animal Science, Rural Development Administration, Cheonan-si, Chungcheongnam-do, Republic of Korea; ^2^Pathologic Diagnostic Laboratory, Animal Disease Diagnostic Division, Animal and Plant Quarantine Agency, Gimcheon-si, Gyeongsangbuk-do, Republic of Korea; ^3^College of Veterinary Medicine, Jeonbuk National University, Iksan-si, Jeollabuk-do, Republic of Korea

**Keywords:** automated monitoring system, blood analysis, *Bos taurus taurus*, haemorrhagic bowel syndrome, necropsy

## Abstract

Jejunal haemorrhage syndrome (JHS) is a sporadic and fatal enterotoxaemic disease in dairy cows associated with acute development and poor prognosis despite treatment. A 5-year-old Holstein cow with no reported pregnancy, three calving numbers, and 303 days in milk presented with hypothermia, discomfort, and inappetence. Anaemia, dehydration, faeces with blood clots, and absence of rumen and bowel movements were observed. We identified the presence of neutrophilia, hyperglycaemia, hypoproteinaemia, azotaemia, hyperlactatemia, hypocalcaemia, hypermagnesemia, hypokalaemia, and hypochloraemia through blood analyses. Necropsy and histopathologic examination revealed a dilated bluish-purple jejunum, blood clots within the jejunum, neutrophil infiltration into the submucosa of the jejunum, and vascular necrosis. Retrospective examination revealed extraordinary patterns of rumination time, activity, rumen mobility, and rumen temperature using biosensors and decreased milk yield. The abnormalities in the affected cow were detected before recognition by farm workers. To the best of our knowledge, this is the first report to examine data from biosensors in a cow with JHS. Our findings suggest that using biometric data may help understand the development of JHS.

Jejunal haemorrhage syndrome (JHS) causes sporadic necrohaemorrhagic enteritis in cattle. The JHS aetiology and pathogenesis remained unelucidated. *Clostridium perfringens* type A and *Aspergillus fumigatus* have been proposed as the causative agents. Shiga toxin-producing *Escherichia coli* was detected in JHS-affected beef cattle that had consumed feed mixed with mycotoxigenic fungi (Cantor 1999; Kirkpatrick et al. 2001; Baines et al. 2011; Elhanafy et al. 2013; Peek et al. 2018; Sharkey and Heinrich 2019). Multiple risk factors affect JHS, including season, parity, days in milk, milk production, feed, and feed intake (Godden et al. 2001; Dennison et al. 2002; Abutarbush and Radostits 2005; Berghaus et al. 2005; Elhanafy et al. 2013; Mamak and Borku 2019).

JHS, an enterotoxaemic disease that progresses acutely in dairy cows, may cause death or poor prognoses despite administering medical and surgical interventions (Elhanafy et al. 2013). Affected cows present with decreased vitality, feed intake, and milk yield. Impaired vessels of the jejunum cause abdominal discomfort, bloody faeces, melena, reduced faecal amount, dehydration, anaemia, tachycardia, and cool extremities (Dennison et al. 2002; Abutarbush and Radostits 2005; Ceci et al. 2006; Braun et al. 2010). Haematological and serum biochemical abnormalities with metabolic alkalosis are common findings in cows with JHS (Dennison et al. 2002; Abutarbush and Radostits 2005; Ceci et al. 2006; Braun et al. 2010; Elhanafy et al. 2013; Mamak and Borku 2019).

In addition, distention and discolouration (dark red to purple) of the jejunum have also been revealed during necropsy. The histopathologic findings of JHS include haemorrhage in the submucosa and inflammatory filtration (Elhanafy et al. 2013; Mamak and Borku 2019).

Biosensors provide information regarding reproduction and health problems by detecting rumination, temperature, activity, and pH; furthermore, biosensors can monitor oestrus, pregnancy, and parturition (Bruinje and Ambrose 2019; Lee and Seo 2021). Since biosensors can detect mastitis, lameness, and metabolic disorders, they offer further understanding of these conditions (Rutten et al. 2013; Bayne et al. 2016; Haladjian et al. 2018; Antanaitis et al. 2020; Alipio and Villena 2022; Antanaitis et al. 2022). However, to the best of our knowledge, no studies have reported data collected using biosensors in dairy cattle with JHS. Therefore, this study aimed to report the biosensor data of a cow with JHS, as well as the findings of the clinical examination, haematological and serum biochemical analyses, and necropsy).

## Case presentation

A 5-year-old milking Holstein cow with 2.75 body condition score (five-point scale with quarter-point divisions), no reported pregnancy, three calving numbers, and 303 days in milk, on November 7, 2022, presented with hypothermia, discomfort, and inappetence. The cow had no history of receiving any treatment for health problems since it was treated for a mild cough at the age of one month (January 2018). The cow was used as an embryo transfer recipient for a Jersey calf and underwent artificial inseminations: however, she did not become pregnant. The cow was raised in a herd that was milked twice a day in the morning and evening using a milking machine (DeLaval, Tumba, Sweden) and fed total mixed rations *ad libitum*, consisting of concentrates, soybean meal, corn silage, alfalfa hay, timothy hay, enzymes, minerals, and vitamin additives. The workers on the farm monitored the quality of feed before making total mixed rations and feeding cows. The other cows in the herd were healthy. The cow was milked until the evening of November 6, 2022, but not on the morning of November 7, 2022, due to a health problem.

**Figure 1:**
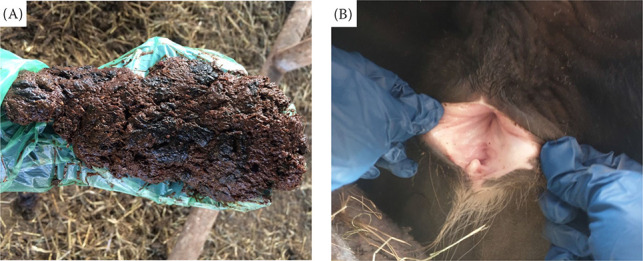
(A) Faeces with blood clot and (B) pale vaginal mucous membrane

On presentation, the cow moved and changed the postures between lateral and sternal recumbency and standing. Dehydration (eyeball recession), hypothermia (rectal temperature 35.9 °C), and tachycardia (102 beats/min) were observed. The respiratory rate (26 breaths/min) was normal. The faeces were firmer than those of others and mixed with a dark black blood clot (Figure 1A). A vacant rectum was identified after examining the faeces. Examination of the vaginal mucous membrane revealed anaemia (Figure 1B). However, auscultation did not reveal rumen contraction, bowel movement, or ping sounds. Blood was collected from the jugular vein to perform the complete blood count and serum biochemistry tests (Procyte Dx^®^ haematology analyser and Catalyst^TM^ Dx chemistry analyser; IDEXX Laboratories, Westbrook, MA, USA); these tests were conducted immediately in a farm laboratory. Moreover, blood gas and electrolyte analyses (EPOC^®^; Siemens Healthcare, Ottawa, ON, Canada) were also performed.

Haematological examination showed low red blood cell count, haematocrit, and haemoglobin, and high mean corpuscular haemoglobin concentration, neutrophil count, and mean platelet volume values (Table 1). Serum biochemistry revealed severe hyperglycaemia and hypocalcaemia, and mild azotaemia and hypermagnesaemia. The total protein and albumin values were low, whereas the lactate levels were high. Blood gas and electrolyte analyses revealed hypokalaemia and hypochloraemia.

**Table 1:** Haematological, serum biochemical, blood gases, and electrolyte parameters in a milking Holstein cow with jejunal haemorrhage syndrome

Haematological parameters	Case (reference range)	Serum biochemical parameters	Case (reference range)	Blood gases and electrolyte parameters	Case (reference range)
RBC count (× 10^12^/l)	4.22 (4.47–9.35)	glucose (mmol/l)	15.71 (3.11–4.88)	pH	7.363 (7.35–7.50)
HCT (%)	19.9 (22.5–39.9)	BUN (mmol/l)	11.07 (3.57–8.92)	pCO_2_ (Pa)	6.32 (5.45–6.65)
Hb (g/l)	71 (74–128)	creatinine (μmol/l)	70.71 (44.20–141.44)	HCO_3_^–^ (mmol/l)	27.1 (24–34)
MCV (fl)	47.2 (40.4–56.4)	phosphorus (mmol/l)	2.23 (1.29–2.78)	tCO_2_ (mmol/l)	27.3 (25–35)
MCH (pg/cell)	16.8 (11.5–18.5)	calcium (mmol/l)	1.20 (2.00–3.00)	Na^+^ (mmol/l)	133 (134–145)
MCHC (g/l)	357 (302–335)	magnesium (mmol/l)	1.47 (0.74–1.23)	K^+^ (mmol/l)	3.2 (3.9–5.3)
RDW (%)	23.5 (20.0–35.9)	total protein (g/l)	58 (62–80)	Cl^–^ (mmol/l)	87 (94–105)
Reticulocyte (× 10^9^/l)	0.8 (0.0–3.9)	albumin (g/l)	24 (25–35)	–	–
WBC count (× 10^9^/l)	16.36 (2.71–17.76)	globulin (g/l)	34 (30–49)	–	–
Neutrophil (× 10^9^/l)	12.28 (0.68–6.94)	ALT (μkat/l)	0.55 (< 1.35)	–	–
Lymphocyte (× 10^9^/l)	2.02 (1.20–10.62)	ALP (μkat/l)	0.89 (0.47–3.89)	–	–
Monocyte (× 10^9^/l)	2.04 (0.02–2.17)	GGT (μkat/l)	0.57 (0–1.45)	–	–
Eosinophil (× 10^9^/l)	0.01 (0.01–1.23)	total bilirubin (μmol/l)	< 1.71 (0.0–11.97)	–	–
Basophil (× 10^9^/l)	0.01 (0.00–0.04)	cholesterol (mmol/l)	3.70 (1.17–5.18)	–	–
PLT count (× 10^9^/l)	210 (147–663)	amylase (μkat/l)	0.38 (0–0.57)	–	–
MPV (fl)	10.1 (5.9–8.2)	lipase (μkat/l)	2.39 (0.50–3.34)	–	–
PDW (fl)	7.2 (6.0–10.1)	lactate (mmol/l)	6.56 (0.56–2.22)	–	–

ALP = alkaline phosphatase; ALT = alanine transaminase; BUN = blood urea nitrogen; Cl^–^ = chloride; GGT = γ-glutamyl transferase; Hb = haemoglobin; HCO_3_^–^ = bicarbonate; HCT = haematocrit; K^+^ = potassium; MCH = mean corpuscular haemoglobin; MCHC = mean corpuscular haemoglobin concentration; MCV = mean corpuscular volume; MPV = mean platelet volume; Na^+^ = sodium; pCO_2_ = partial pressure of carbon dioxide; PDW = platelet distribution width; PLT = platelet; RBC = red blood cell; RDW = red cell distribution width; tCO_2_ = total carbon dioxide; WBC = white blood cell

The reference ranges of the haematological and serum biochemical parameters were provided by the analyser used in the study, except for ALT and lactate. The reference ranges of ALT and lactate were obtained from a book, Large animal internal medicine (Smith 2014). We used the reference range of blood gases and electrolyte parameters provided by Rebhun’s diseases of dairy cattle (Peek et al. 2018)

The body temperature (rectal temperature 36.6 °C) could not be normalised and did not recover despite the intensive and supportive treatments, including intravenous fluid and electrolyte therapy, antibiotics, and nonsteroidal anti-inflammatory drugs. The cow died in the afternoon, and a necropsy was performed immediately.

**Figure 2:**
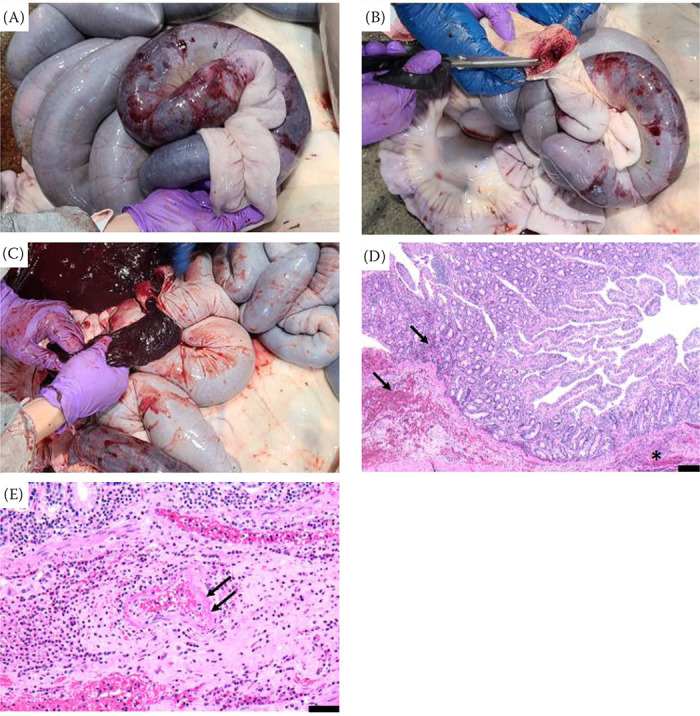
Congested and vacant parts of the jejunum, blood clots in the jejunum, and histopathological findings of JHS in a Holstein cow (A) Congested and dilated jejunum filled with blood clots. (B) Vacant jejunal lumen and the bloody wall of the lumen. (C) Jejunal distention containing blood-clotted intestinal contents. (D) Jejunum. Mucosal and submucosal haemorrhage (black arrows) and infiltration of many neutrophils into the submucosa (asterisk). H&E stain. Scale bar, 200 μm. (E) Jejunum. Magnification of Figure 2D (asterisk) and vascular necrosis (black arrows). H&E stain. Scale bar, 50 μm H&E = haematoxylin and eosin

Necropsy revealed an extensive and dilated bluish-purple jejunum (Figure 2A). No other remarkable findings, except for pale lungs, were observed. The jejunum close to the duodenum was normal and had a fermented total mixed ration, whereas the jejunum near the ileum was pale and without content inside. At the beginning of the incision on the jejunum near the ileum, a bloody lumen wall was identified without any other content except blood (Figure 2B). Blood clots attached to the jejunal lumen wall were observed after finishing the approaching incision to the long dilated bluish-purple jejunum (Figure 2C). Histopathological examination revealed haemorrhage in the mucosa and submucosa of the jejunum and infiltration of many neutrophils into the submucosa (Figure 2D). Vascular necrosis was observed in the submucosa of the jejunum (Figure 2E). No aetiological agents were identified.

**Figure 3:**
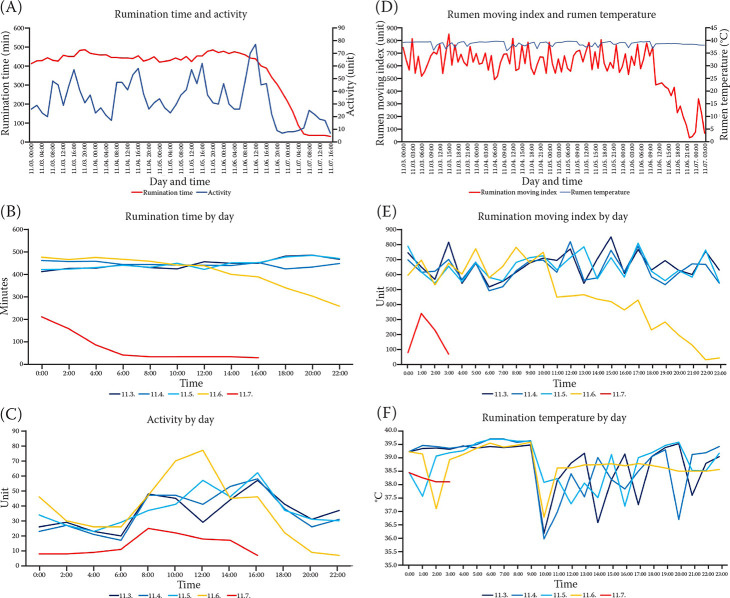
Rumination time, activity, rumen moving index, and rumen temperature of a Holstein cow with JHS The summation of rumination time for the last 24 h and activity for the last 2 h were offered. The rumen moving index and temperature were adjusted to be the summation for an hour and the average for an hour, respectively. (A) Rumination time and activity for the last 5 days before death (November 7). The red line represents rumination time, and the blue line represents activity. (B) Rumination time by day. The rumination time was the summation of time for the cow to ruminate for the past day. (C) Activity by day. (D) Rumen moving index and rumen temperature for the last 5 days before death. The red line represents the rumen moving index, and the blue line represents the rumen temperature. (E) Rumen moving index by day. The rumen moving index was the summation for each hour. (F) Rumen temperature by day. The rumen temperate was the average for each hour

Retrospective examinations were performed using biometric devices (HR-Tag; SCR Engineers Ltd., Netanya, Israel; Smart alyac^®^; Hankook Iot Corp., Gimcheon, Republic of Korea) and an automated milking system (Time for Cows and Astronaut A3; Lely Industries NV, Maassluis, the Netherlands). The summation of rumination time for the last 24 h and activity for the last 2 h were offered. The rumen moving index and rumen temperature were adjusted to be the summation and the average for an hour, respectively. The cow showed abnormalities in the rumination time, activity, rumen moving index, and rumen temperature in the afternoon of November 6, 2022. She routinely spent > 400 mins daily on ruminating; however, the rumination time decreased (< 400 mins) from 14:00 and did not recover (Figure 3A,B). The activity usually ranged from 17 to 67 units. The activity was > 70 units from 10:00 to 12:00 and decreased from 16:00 onwards on November 6 than that from November 3 to 5 (Figure 3A,C). The rumen moving index began to decrease by over 160 from 11:00 on November 6, compared with that at the same time from November 3 to 5 (Figure 3D,E). The rumen temperature fluctuated between 35.97 and 39.70 °C from November 3 to 5 but remained over 38 °C from 11:00 am onwards on November 6 (Figure 3D,F). The evening milk yield on November 5, 2022 was 30% more than that on November 4, 2022. The cow produced a similar amount of milk in the morning, but approximately 30% less in the evening on November 6, 2022, compared with the average milk yield from November 1 to 5, 2022 (Table 2).

**Table 2:** Milk yield before the manifestation onset of jejunal haemorrhage syndrome

Day	Morning milk yield (kg)	Evening milk yield (kg)
3^rd^ November	21.2	9.9
4^th^ November	19.4	10.0
5^th^ November	20.5	13.0
Average from 3^rd^ to 5^th^	20.4 ± 0.9	11.0 ± 1.8
6^th^ November	20.8	7.2

## DISCUSSION AND CONCLUSIONS

JHS causes a radical economic loss to cattle farms, because it is acute, sporadic, and fatal, and with poor prognosis of medical and surgical treatments. JHS has been reported over the last few decades. Although the risk factors and clinical, clinicopathological, and necropsy findings associated with JHS have been identified (Elhanafy et al. 2013; Mamak and Borku 2019), the clinical findings are limited to the onset of clinical examinations. Thus, we investigated the clinical development of JHS using biosensors as well as clinical, clinicopathologic, and necropsy findings.

This case occurred in the autumn, which is the third lactational high-producing-cow-like risk factor for JHS; however, our case was rare wherein approximately 80% of JHS cases occurred during early- and mid-lactation. Similar to previous studies, the cow had hypothermia, discomfort, anorexia, dehydration, anaemia, and bloody and scant faeces and died within 2 days of the onset of clinical signs (Dennison et al. 2002; Abutarbush and Radostits 2005; Ceci et al. 2006; Elhanafy et al. 2013; Mamak and Borku 2019).

Haematological and serum biochemical abnormalities were observed in the present case, and the jejunum was obstructed by blood clots adhering to the jejunum wall. Interstitial fluid decreases the red blood cell count, haematocrit, haemoglobin, and plasma protein concentrations within a few hours after the onset of haemorrhage (Latimer 2011). Haemorrhage in JHS may have caused the decrease in red blood cell count, haematocrit, haemoglobin, and serum albumin concentration in cows. Increased blood urea nitrogen concentration without increasing serum creatinine concentration can be attributed to JHS due to blood breakdown in the intestine secondary to small bowel haemorrhage (Dennison et al. 2002; Ceci et al. 2006; Latimer 2011).

Neutrophils may be associated with JHS and affected cows show neutrophilia and hyperglycaemia, which are common findings in cows with JHS due to stress-dependent responses (Dennison et al. 2002; Abutarbush and Radostits 2005; Ceci et al. 2006; Braun et al. 2010). Moreover, the infiltration of neutrophils into the submucosa of the jejunum was identified, similar to previous studies (Abutarbush et al. 2004; Owaki et al. 2015).

Metabolic alkalosis with hypokalaemia and hypochloraemia are common findings in JHS due to abomasal secretions and mechanical and functional proximal bowel obstruction (Dennison et al. 2002; Abutarbush and Radostits 2005; Ceci et al. 2006; Braun et al. 2010). The blood pH in this case was close to acidosis but within the reference range. Increased lactate levels may contribute to the pH change and be associated with JHS due to endotoxaemia, cellular hypoxia, and hypovolaemia following haemorrhage (Giertzuch et al. 2020). Hypocalcaemia and hypermagnesemia are common findings in JHS (Elhanafy et al. 2013). The proteases containing calcium produced by multiplying *C. perfringes* type A could decrease the calcium availability in the gastrointestinal tract, and metabolic alkalosis may contribute to decreased parathyroid hormone responsiveness (Elhanafy et al. 2013). However, this assumption cannot be applied to all cases of JHS. The aetiologic agents of JHS have not yet been determined (Elhanafy et al. 2013; Mamak and Borku 2019), and *C. perfringes* type A was not detected in this study. Hypocalcaemia may be attributed to the massive blood clot in the intestine, wherein serum calcium is used for coagulation. However, the cause of the massive blood clot in the intestine remains unclear in this study. Cattle have more magnesium in erythrocytes than that in the plasma (or serum). Blood breakdown may contribute to hypermagnesemia. In addition, the parathyroid hormone caused by hypocalcaemia may increase serum magnesium levels by absorbing the magnesium in the intestine and resorption in the kidney and bone (Scott and Stockham 2013).

Biosensors are used to detect health events in cows. During the oestrus day, cows show higher activity with similar patterns and less rumination time than a few days before oestrus (Jeon et al. 2022). Sensing data are different according to each disorder of cows. Hypocalcaemia is associated with decreased rumination time, activity, rumen temperature, and milk yield during the first 1 to 3 days after calving (Tsai et al. 2021). Lame cows showed decreased rumination time over 2 weeks before the onset of clinical signs than healthy cows (Antanaitis et al. 2021). In *Anaplasma* infection, rumination time decreases by over 30% from 4 days before the onset of manifestation (Teixeira et al. 2022). In endotoxin-induced clinical mastitis, cows show no difference in the total daily rumination time before and after toxin infusion since they spend a longer time ruminating to compensate for a decrease in rumination time in the hours following the toxin infusion (Fitzpatrick et al. 2013). To the best of our knowledge, no study has been conducted on JHS using biosensing data. In our case, the rumination time, activity, rumen moving index, and rumen temperature measured using biosensors showed different patterns and abnormalities about one day before visual recognition by farm workers in this case. The cow had continuously decreasing rumination time, which was less than the usual rumination time. In addition, the activity was higher than usual for a few hours and then lower than usual. The rumen moving index and rumen temperature usually fluctuate a few hours after feeding. Rumen temperature may be associated with drinking water, considering cows drink water after eating feed. After the onset of JHS, this cow had a lower rumen moving index and relatively constant rumen temperature. The affected cow may not drink water due to JHS-related pain. Decreased rumination time, activity, and rumen temperature are associated with hypocalcaemia (Tsai et al. 2021). Thus, the decreased rumination time, activity, and rumen temperature in this cow with JHS may also be attributed to hypocalcaemia. The rumination time, activity, rumen mobility, and rumen temperature measured using biosensors may be used as indicators of JHS. It may be common for affected cows to exhibit decreased milk yield after showing abnormalities in rumination time, activity, rumen mobility, and rumen temperature. However, it is extraordinary for a cow in late lactation to produce > 30% more milk than the previous day. Further studies are required to investigate the effects of JHS on cows in terms of data from biosensors and milk yield.

Drastic decreases in rumination time, activity, rumen mobility, rumen temperature, and milk yield obtained from biosensors and milking systems in a day may provide early alerts regarding health problems, including JHS, in a particular cow, indicating the need for further examinations. Moreover, it can help understand the development of JHS. However, further studies should be conducted to establish the association between biometric data and JHS for the early detection of JHS.
